# Assessing Whether Alpha-Tubulin Sequences Are Suitable for Phylogenetic Reconstruction of Ciliophora with Insights into Its Evolution in Euplotids

**DOI:** 10.1371/journal.pone.0040635

**Published:** 2012-07-10

**Authors:** Zhenzhen Yi, Laura A. Katz, Weibo Song

**Affiliations:** 1 Key Laboratory of Ecology and Environment Science in Guangdong Higher Education, College of Life Science, South China Normal University, Guangzhou, China; 2 Laboratory of Protozoology, Institute of Evolution & Marine Biodiversity, Ocean University of China, Qingdao, China; 3 Department of Biological Sciences, Smith College, Northampton, Massachusetts, United States of America; University of Oxford, United Kingdom

## Abstract

The current understanding of ciliate phylogeny is mainly based on analyses of a single gene, the small subunit ribosomal RNA (SSU-rDNA). However, phylogenetic trees based on single gene sequence are not reliable estimators of species trees, and SSU-rDNA genealogies are not useful for resolution of some branches within Ciliophora. Since congruence between multiple loci is the best tool to determine evolutionary history, we assessed the usefulness of alpha-tubulin gene, a protein-coding gene that is frequently sequenced, for ciliate phylogeny. Here, we generate alpha-tubulin gene sequences of 12 genera and 30 species within the order Euplotida, one of the most frequently encountered ciliate clades with numerous apparently cosmopolitan species, as well as four genera within its putative sister order Discocephalida. Analyses of the resulting data reveal that: 1) the alpha-tubulin gene is suitable phylogenetic marker for euplotids at the family level, since both nucleotide and amino acid phylogenies recover all monophyletic euplotid families as defined by both morphological criteria and SSU-rDNA trees; however, alpha-tubulin gene is not a good marker for defining species, order and subclass; 2) for seven out of nine euplotid species for which paralogs are detected, gene duplication appears recent as paralogs are monophyletic; 3) the order Euplotida is non-monophyletic, and the family Uronychiidae with sequences from four genera, is non-monophyletic; and 4) there is more genetic diversity within the family Euplotidae than is evident from dargyrome (geometrical pattern of dorsal “silverline system” in ciliates) patterns, habit and SSU-rDNA phylogeny, which indicates the urgent need for taxonomic revision in this area.

## Introduction

Current studies on the relationships within the phylum Ciliophora are almost exclusively based on SSU-rDNA phylogenies [1]–[7]. These single gene analyses provided resolution for a number of important questions on the phylogenetic relationships within this group, but there are problems. The overall picture emerging from these studies confirmed the monophyly of most classes defined by morphological criteria; however, relationships among these classes vary with different taxon sampling (for example [3], [8]–[10]). Moreover, while some previous investigations based on SSU-rDNA alone resolved assignments of some taxa with ambiguous morphological classification (for example [4], [11], [12]), relationships within some orders/families containing a large number of taxa remain problematic [6], [13].

In recent years, other gene markers, including LSU-rDNA gene, ITS region, tubulins, phosphoglycerate kinase, actin, DNA Polymerase α, Hsp 70, etc., have been used to reconstruct ciliate phylogenies [14]–[22]. Traditionally, protein gene markers are considered more suitable alternatives to SSU-rDNA than LSU-rDNA gene and ITS region for two reasons. First, protein markers are less sensitive to differences in compositional bias, which can lead to artifacts in tree construction [23]–[25]. In addition, sequences of multiple unlinked loci have different histories, as opposed to linked SSU-rDNA, LSU-rDNA, ITS regions, and are necessary to estimate species trees [26]. However, protein-coding genes can possess paralogs that might bias phylogenetic trees [27], [28]. Among these protein-coding genes, alpha-tubulin is one of the mostly used gene makers for ciliated phylogeny [8], [11], [13], [15], [20], [21], [29], and its duplication in ciliates has been previously studied only with sparse taxon sampling [15], [30]. Therefore, alpha-tubulin is a promising candidate for testing whether protein-coding genes are suitable for phylogeny construction of ciliates.

Previous alpha-tubulin phylogenies showed that most classes could be well distinguished with high support [8], [15], [20], while subclasses appeared to be non-monophyletic [13], [21], [29]. In our recent study [13], we characterized alpha-tubulin gene from 15 genera covering all families of the order Urostylida, but we were unable to determine if alpha-tubulin gene is suitable for classification of lower level taxa since urostylid families are not well defined morphologically [31], [32] and their monophyly is rejected by both SSU-rDNA and alpha-tubulin phylogenies. Therefore, a group with well-defined families or genera is needed to test the ability of alpha-tubulin to resolve phylogeny for lower level taxa. The order Euplotida, one of the most frequently encountered ciliate clades with numerous putatively cosmopolitan species [33]–[37], is a good choice because most morphological families within this order are recovered robustly in SSU-rDNA analyses [38]–[40].

Here, we increase sampling of alpha-tubulin gene sequences from 30 taxa within the order Euplotida, including multiple morphospecies from four out of five families, as well as four species of its putative sister order Discocephalida. Our main aims are to: 1) assess the suitability of alpha-tubulin for circumscribing lower level taxa; 2) estimate phylogenetic relationships within the order Euplotida using two-gene combined, SSU-rDNA and alpha-tubulin trees; and 3) characterize patterns of molecular evolution among euplotid alpha-tubulin paralogs.

## Results

### Different Species with Same Amino Acid Sequences

Although species contained diverse alpha-tubulin sequences, we found that some euplotid species (e.g. *Uronychia multicirrus* and *U. sinica*; *Euplotes* sp.-GZJJM2009121510, *Euplotoides parawoodruffi* and *Euplotopsis* sp.-GZJJM2009121508; *Euplotopsis encysticus* and *Euplotes* cf. *antarcticus*) share identical amino acid sequences, revealing the high level of functional constraint on this protein (Fig. S1). Alpha-tubulin gene sequences of *U. multicirrus* and *U. sinica* are different from each other at 56 sites, all of which are third codon positions (Fig. S1). Similarly, 69 residues, 3 first codon positions and 66 third codon positions, are different between alpha-tubulin gene sequences of *Euplotopsis encysticus* and *Euplotes* cf. *antarcticus* (Fig. S1). There are totally 124 polymorphic nucleotide sites between *Euplotes* sp.-GZJJM2009121510, *Euplotoides parawoodruffi* and *Euplotopsis* sp.-GZJJM2009121508. And among these sites, 11 are in first codon positions, only one is in second codon position, and the remaining 112 sites are in third codon positions (Fig. S1).

### Intraspecific Variation

Multiple clones have been sequenced from ten species and two populations of the morphospecies *Diophrys parappendiculata* and *Euplotes sinicus* are sampled (Table 1). No paralogs are detected in two populations of *D. parappendiculata*. Only one sequence was found in *E. sinicus* population II, while two paralogs are present in population I, with the first being identical to the sequence of population I. (Table 2).

**Table 1:** Euplotid Species for Which Alpha-Tubulin Genes Were Sequenced in the Present Work.

Taxa	Sampling localityLocation	DNA sample	GenBank No.	Taxa	Sampling localityLocation	DNA sample	GenBank No.
***Aspidisca aculeata***	Daya Bay (22°43′N; 114°32′E), Guangdong	one cell	JQ736724	***Euplotes sinicus*** ** popI**	Qingdao (36°03′N; 120°20′E), Shandong	one cell	JQ73674-JQ736748
***Aspidisca leptaspis*** ** P1-P3**	Qingdao (36°03′N; 120°20′E), Shandong	20 cells	JQ736687-JQ736691	***Euplotes sinicus*** ** popII**	Daya Bay (22°43′N; 114°32′E), Guangdong	20 cells	JQ736734,
***Aspidisca magna***	Gaoqiao (21°31′N; 109°45′E), Guangdong	one cell	JQ736722	***Euplotes*** ** sp.-GZJJM2009121510**	Zhanjiang (21°27′N; 110°35′E), Guangdong	one cell	JQ736721
***Aspidisca orthopogon*** ** P1-P2**	Qingdao (36°03′N; 120°20′E), Shandong	3 cells	JQ736740, JQ736741	***Euplotoides parawoodruffi***	Daya Bay (22°43′N; 114°32′E), Guangdong	10 cells	JQ736737
***Aspidisca steini***	Gaoqiao (21°31′N; 109°45′E), Guangdong	2 cells	JQ736723	***Euplotopsis encysticus***	Daya Bay (22°43′N; 114°32′E), Guangdong	4 cells	JQ736725
***Apodiophrys ovalis*** ** P1-P3**	Dameisha (22°59′N; 114°30′E), Guangdong	one cell	JQ736709-JQ736717	***Euplotopsis raikovi***	Qingdao (36°03′N; 120°20′E), Shandong	12 cells	JQ736749
***Diophryopsis hystrix*** ** P1-P3**	Qingdao (36°03′N; 120°20′E), Shandong	one cell	JQ736694-JQ736698	***Euplotopsis*** ** sp.-GZJJM2009121508**	Zhanjiang (21°27′N; 110°35′E), Guangdong	one cell	JQ736720
***Diophrys apoligothix***	Qingdao (36°03′N; 120°20′E), Shandong	several cells	JQ736742	***Gastrocirrhus monilifer***	Qingdao (36°03′N; 120°20′E), Shandong	4 cells	JQ918351
***Diophrys scutum*** ** P1-P2**	Qingdao (36°03′N; 120°20′E), Shandong	5 cells	JQ736692, JQ736693	***Gastrocirrhus*** ** sp.-GZCXM2008122201**	Daya Bay (22°43′N; 114°32′E), Guangdong	8 cells	JQ736708
***Diophrys parappendiculata*** ** popI**	Daya Bay (22°43′N; 114°32′E), Guangdong	9 cells	JQ736739	***Heterodiophrys zhui***	Dameisha (22°59′N; 114°30′E), Guangdong	several cells	JQ736718
***Diophrys parappendiculata*** ** popII**	Daya Bay (22°43′N; 114°32′E), Guangdong	5 cells	JQ918349	***Leptoamphisiella vermis***	Qingdao (36°03′N; 120°20′E), Shandong	several cells	JQ736707
***Discocephalus rotatorius***	Dameisha (22°59′N; 114°30′E), Guangdong	several cells	JQ736719	***Moneuplotes minuta***	Qingdao (36°03′N; 120°20′E), Shandong	one cell	JQ736750
***Euplotes*** ** cf. ** ***antarcticus***	Daya Bay (22°43′N; 114°32′E), Guangdong	30 cells	JQ736735	***Paradiophrys zhangi***	Qingdao (36°03′N; 120°20′E), Shandong	4 cells	JQ736743, JQ736744
***Euplotes balteatus***	Qingdao (36°03′N; 120°20′E), Shandong	20 cells	JQ736702	***Pseudoamphisiella elongate***	Qingdao (36°03′N; 120°20′E), Shandong	4 cells	JQ918350
***Euplotes charon***	Qingdao (36°03′N; 120°20′E), Shandong	one cell	JQ736751	***Prodiscocephalus borrori***	Qingdao (36°03′N; 120°20′E), Shandong	one cell	JQ918352
***Euplotes neaplolitanus*** ** P1-P3**	Daya Bay (22°43′N; 114°32′E), Guangdong	4 cells	JQ736726-JQ736733	***Uronychia multicirrus***	Daya Bay (22°43′N; 114°32′E), Guangdong	10 cells	JQ736738
***Euplotes petzi***	Qingdao (36°03′N; 120°20′E), Shandong	3 cells	JQ736703-JQ736705	***Uronychia sinica***	Mangrove in Shenzhen (22°37′N; 114°04′E 1), Guangdong	15 cells	JQ736736
***Euplotes rariseta***	Qingdao (36°03′N; 120°20′E), Shandong	31 cells	JQ736749	***Uronychia*** ** sp.-SXL2007102501**	Hangzhou (30°16′N; 120°10′E 1), Zhejiang	8 cells	JQ918348

**Table 2:** Intraspecific Distances Between/Among α-Tubulin Clones and Between/Among Paralogs.

Taxa	Comparisonof Paralog(s)	Clone names	N	R/S	*d*	*dA*
***Apodiophrys ovalis***	1	Clone 1–5	5	1.6/2	0.337±0.109	0.927±0.328
	2	Clone 6	1	–	–	–
	3	Clone 7–9	3	0.7/2	0. 249±0. 135	0.180±0.176
	1, 2, 3			3.3/91.7#	10.265±1.106	0.398±0.178
***Aspidisca leptaspis***	1	Clone 1	1	–	–	–
	2	Clone 2–4	3	0/0	0.000	–
	3	Clone 5	1	–	–	–
	1, 2, 3			10.3/55#	6.317±0.789	1.948±0.596
***Aspidisca orthopogon***	1	Clone 1	1	–	–	–
	2	Clone 2	1	–	–	–
	1, 2			7/51#	6.255±0.855	1.785±0.680
***Diophryopsis hystrix***	1	Clone 1	1	–	–	–
	2	Clone 2	1	–	–	–
	3	Clone 3, 4	2	0/7	0.658±0.241	0.000±0.000
	4	Clone 5	1	–	–	–
	1, 2, 3, 4			7/64.3#	7.452±0.859	0.911±0.421
***Diophrys scutum***	1	Clone 1	1	–	–	–
	2	Clone 2	1	–	–	–
	1, 2			5/18	2.183±0.450	0.808±0.414
Diophrys parappendiculata*	1	Clone 1, 2	2	0/0	0.000	0.000±0.000
***Euplotes neaplolitanus***	1	Clone 1	1	-	–	–
	2	Clone 2, 3	2	1/3	0.468±0.227	0.270±0.260
	3	Clone 4–8	5	1.2/0.4	0.150±0. 074	0.324±0.180
	1, 2, 3			6/69.3#	7.988±0.852	1.284±0.540
***Euplotes petzi***	1	Clone 1–3	3	3/10.3	0.792±0.245	0.878±0.381
***Euplotes sinicus*** **	1	Clone 1, 2, 5	3	3/5	0.784±0.176	0.188±0.019
	2	Clone 3, 4	2	2/5	0.729±0.270	0.545±0.365
	1, 2			2/24#	3.763±0.564	1.219±0.524
***Paradiophrys zhangi***	1	Clone 1, 2	2	3/5	0.999±0.345	0.718±0.479

NOTE.**-** *N,* number of clones; *d*, number of nucleotide substitutions per site calculated using Tamura-Nei model; *dA*, number of amino acid substitutions per site calculated using Dayhoff model; R/S, number of replacement site substitutions/number of synonymous substitutions among clones.

# Fixed between paralogs.

** *Euplotes sinicus* population I: Clone 1–4; *E. sinicus* population II: Clone 5.

The intraspecific variation among putative orthologs for these species ranges between 0% (*Aspidisca leptaspis* P2, *Paradiophrys zhangi*) and 0.792% (*Euplotes petzi*) (Table 2). Among these ten species, paralogs appear absent in four: *Euplotes petzi*, *Diophrys parappendiculata*, *Paradiophrys irmgard* and *P*. *zhangi*. For these, average pairwise difference among clones within each of these taxa is low (0.000–0.999%; Table 2). Ratios of replacement substitutions to silent substitutions are 3/10.3, 0/0, 0/0, and 3/5 for *E. petzi*, *D. parappendiculata*, *P. irmgard*, and *P*. *zhangi*, respectively. Paralogs are detected for remaining six species: *Apodiophrys ovalis*, *Aspidisca leptaspis*, *A. orthopogon*, *Euplotes neapolitanus*, *Diophrys scutum* and *Diophryopsis hystrix* (Table 2). Among these six species, there are more synonymous site substitutions than replacement substitutions both within paralogs and fixed between paralogs. Synonymous substitutions appear less frequent than replacement substitutions for *E. neapolitanus* paralog P3 (1.2/0/4), but the small numbers here suggest that experimental error may be contributing factor (Table 2). The average pairwise amino acid difference among clones of a specific paralog is 0.000% (*Aspidisca leptaspis* P2, *Diophrys parappendiculata* and *P. irmgard*) to 0.792% (*E*. *petzi* P1), and that between/among paralogs of a specific species is from 0.398% (*Apodiophrys ovalis*) to 1.948% (*Aspidisca leptaspis*) (Table 2).

**Figure 1:**
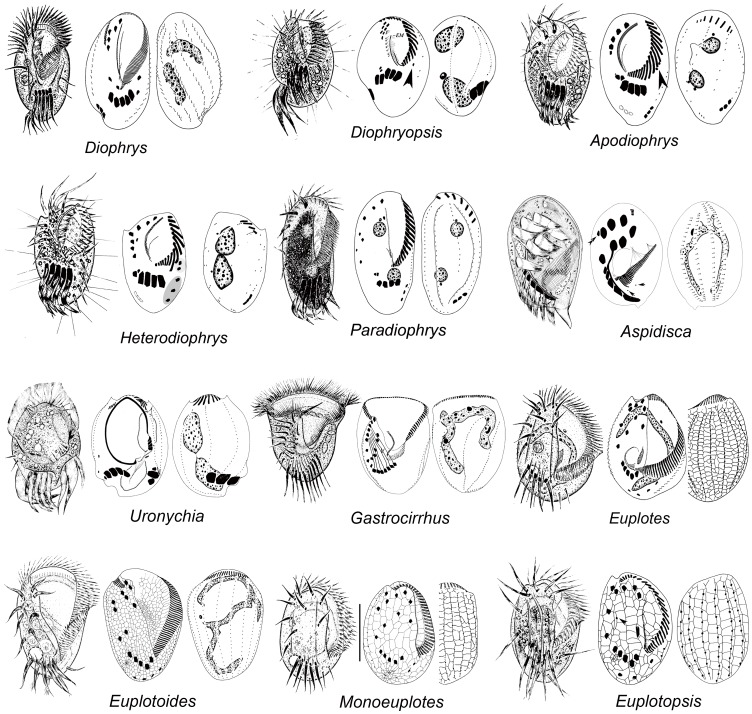
Representative euplotid species from live material and after protargol impregnation.

### Phylogenetic Trees Inferred from Alpha-Tubulin Nucleotide (Atub_n74, Atub_n52) and Amino acid Sequences (Atub_aa)

**Figure 2:**
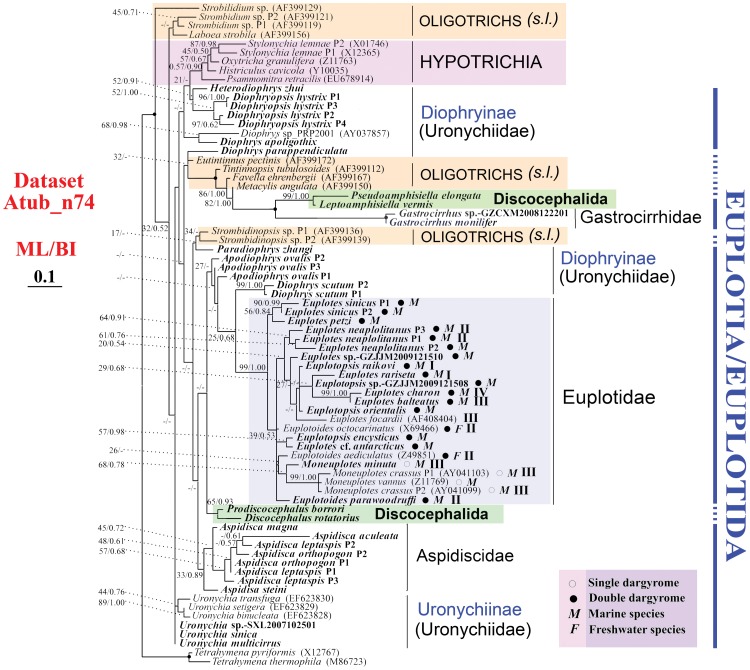
Best tree of the Spirotrichea inferred by Maximum likelihood of Dataset Atub_n74. Species newly sequenced in the present study are shown in bold type. Bootstrap values for branches of the ML tree and posterior probability values for BI tree, respectively, are given on nodes. Fully supported (100%/1.00) branches are marked with solid circles. The scale bar corresponds to 10 substitutions per 100 nucleotide positions. Dargyrome patterns and natural habitats are given after species name of euplotids by symbols. Clades I-IV for euplotids were designated according to Petroni [42] and Yi et al. [38].

We analyzed three different alpha-tubulin datasets: nucleotides from 74 taxa (Atub_n74), nucleotides from a subset of 52 taxa (Atub_n52, only the paralog with shortest branch length in Atub_n74 is selected) and 70 amino acid sequences (Atub_aa). Within the class Spirotrichea, Hypotrichia appears as monophyletic in all analyses (Figs. 2, S2 and S3). For example, *Stylonychia*, *Oxytrichia*, *Histriculus* and *Psammomitra* always fall into the same clade. Oligotrichia is shown to be monophyly in Figure 2 (Atub_n74) and Figure S2 (Atub_aa). The other subclass/order level taxa, i.e. Choreotrichia, Euplotia/Euplotida and Discocephalida, are not monophyletic, which may reflect limited taxon sampling (Figs. 2, S2, S3, Table 3).

**Table 3:** Support for Major Clades of Spirotrichean Species in Analyses Based on Five Datesets.

	Alpha-tubulin			Two-gene combined (52), Atub-SSU	SSU-rDNA (52), SSU
	Nucleotide (74), Atub_n74	Nucleotide (52), Atub_n52	Amino acid (70), Atub_aa		
**Supported clades**					
Aspidiscidae/*Aspidisca*	33%/0.89	40%	45%/0.79	98%	40%
Euplotida/Euplotidae	99%/1.00	98%	66%/0.66	100%	98%
Gastrocirrhidae/*Gastrocirrhus*	100%/1.00	100%	1.00/100%	100%	100%
*Moneuplotes*	68%/0.78	nm	66%/0.99	99%	nm
**Weak hypothesis**					
Choreotrichia	nm	nm	nm	95%	93%
Discocephalida	nm	nm	nm	nm	nm
Hypotrichia	57%/0.90	52%	37%/nm	nm	nm
Oligotrichia	nm	nm	27%/0.98	95%	91%
Uronychiidae	nm	nm	nm	nm	nm
*Diophrys*-complex	nm	nm	nm	nm	nm
*Euplotes*	nm	nm	nm	nm	nm
*Euplotoides*	nm	nm	nm	100%	100%
*Euplotopsis*	nm	m	nm	nm	nm
*Uronychia*	nm	nm	nm	100%	100%
**Dataset statistics**					
Number of lineages	59	49	44	49	48

NOTE.**-**nm = nonmonophyletic.

Within the order Euplotida, monophyly of the family Gastrocirrhidae is supported with highest bootstrap values in all alpha-tubulin trees (Figs. 2, S2 and S3), though only two species are sequenced. Similarly, the family Euplotidae appears to be monophyletic with variable support values (66%–99% ML, 0.66–1.00 BI; Figs. 2, S2, S3). Within this family, *Euplotes*, *Euplotoides* and *Euplotopsis* are always non-monophyletic, and monophyly of the genus *Moneuplotes* is found only in Datasets Atub_n74 (Fig. 2) and Atub_aa (Fig. S2).

Relationships among species in the family Euplotidae do not always corresponding to dargyrome patterns or natural habitats. For example, species possessing single dargyrome always cluster with each other, while those of double dargyrome fall into several clades (Figs. 2, S2, S3). Three stable clades (Clade I-III) found in previous phylogenetic analyses inferred from SSU-rDNA sequences [38], [41], [42] are not recovered here. In our analyses (Figs. 2, S2, S3), monophyly of members of Clade I (*Euplotopsis raikovi*, *Euplotes rariseta*) is never found. Species of Clade II (*Euplotoides parawoodruffi*, *E. octocarinatus*, *E. aediculatus*) fall into different clades, and *Euplotes focardii* is always apart from the other three species of Clade III (*Moneuplotes minuta*, *M. crassus*, *M. vannus*) in all our alpha-tubulin trees (Figs. 2, S2, S3).

Taxa among the family Aspidiscidae group together but with low support values (33%–45% ML, 0.79–0.89 BI) (Figs. 2, S2, S3). Within this clade, *Aspidisca steini* diverges first from remaining species, followed by *A. magna* (Figs. 2, S2, S3). The family Uronychiidae is the only non-monophyletic family out of two families with sequenced samples from multiple genera, and the monophyly of it is only shown in Dataset Atub_n52 (Fig. S3); In contrast, *Uronychia setigera*, *U. transfuga* and *U. binucleata* always cluster together (Figs. 2, S2, S3). The *Diophrys*-complex (viz. *Apodiophrys*, *Diophrys*, *Diophryopsis* and *Heterodiophrys*) species appear in several clades, and their relationships are distinct in trees based on different datasets (Figs. 2, S2, S3).

Within the order Discocephalida, two families (viz. Pseudoamphisiellidae and Discocephalidae) are included. The Pseudoamphisiellidae (*Pseudoamphisiella* and *Leptoamphisiella*) form a monophyletic group as do the Discocephalidae (*Discocephalus* and *Prodiscocephalus*), indicating the monophyly of these two families. However, sister relationship between these two families is never recovered (Figs. 2, S2, S3).

In our phylogenetic trees (Figs. 2, S2, S3), nine species, viz. Apodiophrys ovalis, Aspidisca leptaspis, A. orthopogon, Diophryopsis hystrix, Diophrys scutum, Euplotes neapolitanus, E. sinicus, Strombidinopsis sp., Stylonychia lemnae, have paralogs. Among them, paralogs of Aspidisca leptaspis and A. orthopogon do not cluster together in analyses of Atub_n74 (Fig. 2) and Atub_aa (Fig. S2), respectively; however, they are always in Aspidisca-clade.

### Phylogenetic Analyses Inferred from Two-Gene Combined Dataset (ATUB-SSU) and SSU-rDNA Dataset (SSU)

**Figure 3:**
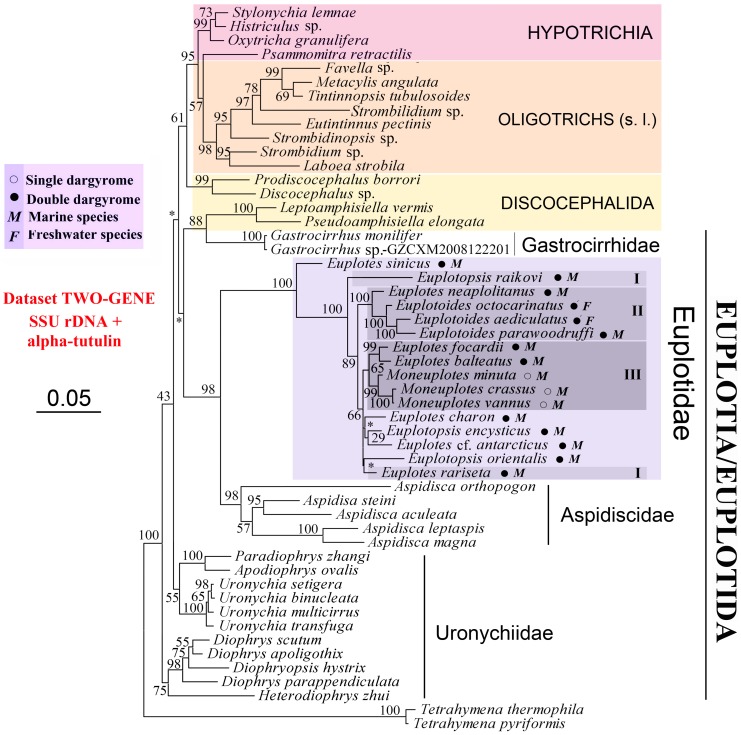
Best tree of the Spirotrichea inferred by two-gene combined sequences (Atub-SSU). Bootstrap values for branches of the ML tree is given on nodes. The scale bar corresponds to 5 substitutions per 100 nucleotide positions. Dargyrome patterns and natural habitats are given after species name of euplotids by symbols. Clades I-IV for euplotids were designated according to Petroni [42] and Yi et al. [38].

Topologies of two-gene combined tree (Fig. 3) and SSU-rDNA tree (Fig. S4) are nearly the same. There are several notable differences from analyses of alpha-tubulin alone (Fig. 2, Figs. S2, S3) including: 1) the monophyly of Oligotrichia and Choreotrichia; and 2) the non-monophyly of Hypotrichia and Discocephalida. Species of Euplotida cluster into a clade in SSU-rDNA tree with no support (Fig. S4), and fall into different clades in two-gene combined tree (Fig. 3). Similar to the alpha-tubulin analyses (Figs. 2, S2, S3), three out of four euplotid families, i.e. Euplotidae, Gastrocirrhidae and Aspidiscidae, are monophyletic in SSU-rDNA (Fig. S4) and two-gene combined trees (Fig. 3), though only several species of one genus are sequenced in the last two families, respectively. As shown in the alpha-tubulin trees (Figs. 2, S2, S3), Clade I as determined by previous investigations [38], [41], [42] does not appear in two-gene combined tree (Fig. 3) and SSU-rDNA tree (Fig. S4). However, *Euplotes focardii*, *E. balteatus*, two species not included in Petroni et al. [42] and Yi et al. [38], and three *Moneuplotes* species group together and form Clade III (Figs. 3, S4).

Nodes of two-gene combined tree (Fig. 3) are better supported than SSU-rDNA tree (Fig. S4). There are 43 and 41 supported nodes (Bootstrap values >50%) in two-gene combined tree (Fig. 3) and SSU-rDNA tree (Fig. S4), respectively. Among them, support values of 28 nodes for these two trees are more than 90%, but more nodes are fully supported in two-gene combined tree (13 for combined tree *vs.* 10 for SSU-rDNA tree). Moreover, the two-gene combined tree (Fig. 3) posses more monophyletic taxa as predicted by morphology (e.g. spirotrichean subclasses and euplotid families, genera) than other trees (Table 3).

## Discussion

### Is Alpha-Tubulin a Suitable Marker for Inferring Ciliate Phylogeny?

The topologies of trees inferred from of ciliate proteins may be confounded by the many paralogs present in these lineages [14], [18] However, in the present investigation, alpha-tubulin gene paralogs of euplotid species are not very divergent from one another, and only paralogs of two of the nine species are non-monophyletic (Figs. 2, S2, S3). Similarly, with samples of five species from three ciliate classes, Israel et al. [15] also found that alpha-tubulin gene paralogs in any given taxon appear to be most closely related to each other or to a sequence from a congener than to others These data indicate that only recent paralogs of alpha-tubulin are retained, and thus gene duplication may not pose a substantial problem in defining ciliate clades [30]. However, alpha-tubulin is not always a good marker for studying relationships at the level of species or below given the high level of amino acid conservation among sequences (Fig. S1). Moreover, it is possible that a combination of gene duplication followed by concerted evolution and differential extinction of some alpha-tubulin paralogs has obscured the evolutionary history in some part of the ciliate tree [15].

The best way to evaluate the quality of one gene marker for tree construction is to look for its congruence with species tree inferred by morphology [14] and by other gene markers. We follow the criterion as a modified one given by Budin and Philippe [14], which is to assess the recovery of the monophyletic groups unquestionably supported by both morphology and SSU-rDNA trees. The monophyly of the family Euplotidae recovered by SSU-rDNA trees is consistently reconstructed in our three alpha-tubulin trees. In recent study, only three out of eight genera (*Moneuplotes*, *Gastrocirrhus* and *Aspidisca*), with alpha-tubulin gene sequences from several species, are monophyletic (Figs. 2, S2, S3). And for the other five genera, only species within *Uronychia* appear to be monophyletic. Same situation occurs in SSU-rDNA analyses [38]–[40], [43]–[45]. Therefore, according to the important criterion of accepted monophyletic groups, the reliability of alpha-tubulin is comparable to that of SSU-rDNA at the genus and family levels.

In the present investigation, only the subclass Hypotrichia, which contains only four genera, is monophyletic in three alpha-tubulin gene trees (Figs. 2, S2, S3). However, with more samples of alpha-tubulin gene from the Hypotrichia, monophyly of Hypotrichia was rejected by previous investigation [13]. For the other four subclasses for which we have sufficient taxon sampling, Oligotrichia is monophyletic (Figure 2, Atub_n74; Figure S2, Atub_aa), and others are not monophyletic. Therefore, alpha-tubulin might not be a good gene marker for examine relationships among high level taxa.

### Ambiguous Assignment of Discocephalida

The phylogenetic position of the sister taxa *Prodiscocephalus* and *Discocephalus* is not stable in our analyses as that their position varies in different trees (Figs. 2, 3, S2, S3, S4). This corresponds to the variable classification schemes for this clade based on morphological and morphogenetic characters [31], [46]–[52]. For example, *Prodiscocephalus* and *Discocephalus* were regarded as genus-complex, family, suborder and order in previous investigations, and were also considered as members of Euplotidae/Euplotida [31], [46], [49], Sporadotrichina, Hypotrichida [48], Oxytrichia [51], [52], Stichotrichia [50] and so on. A relationship between pseudoamphisiellids and discocephalids is only revealed in SSU-rDNA tree (Fig. S4) and the sister relationship of these two groups is not rejected by AU test of Atub_aa (*p* = 0.152) and Atub-SSU (*p* = 0.241).

### Phylogenetic Relationships within the Order Euplotida

Multi-gene analyses are proving useful as a means of placing some taxa within phylogenetic trees where morphological evidence and single gene analyses have not been successful [53]–[56]. Our results also show that two-gene combined tree is better than single gene trees for most clades (Table 3). However, relationship among four euplotid genera was not resolved by any of our analyses, including two-gene combined tree (Figs. 2, 3, S2, S3, S4). Inclusion of more taxa, especially species within the family Certesiidae, coupled with more genes are likely necessary to resolve sister relationships among euplotid families.

All six genera of the family Uronychiidae have been sequenced in the present study, revealing that this family is not monophyletic (Figs. 2, 3, S2, S3, S4). This result is consistent with some previous investigations inferred from SSU-rDNA sequences [45], [57], [58], though in some other SSU-rDNA trees this family appears monophyletic [38]–[40], [44]. Therefore, it is too early to infer whether this family should be further defined before more gene information is available. Similarly, the *Diophrys*-complex contains five genera (*Diophrys*, *Diophryopsis*, *Paradiophrys*, *Heterodiophrys* and *Apodiophrys*) but due to variable positions in different trees, it is difficult to infer their related relationships. However, similar to previous SSU-rDNA investigations [38]–[40], [44], the genus *Diophrys* seems to be non-monophyletic.

The family Euplotidae is composed of *Euplotes*-complex, and was divided into several genera or groups based on different morphological characters [34], [59], [60] or SSU-rDNA trees [38], [41], [42]. However, these classifications are not consistent with one another. For example, based on cortical structure, endosymbionts, morphometric data, morphogenetic patterns, and ecology, *Euplotes*-complex was separated into four genera (i.e. *Euplotes*, *Euplotopsis*, *Euplotoides* and *Moneuplotes*) by Borror and Hill [34]. Previous SSU-rDNA trees [38], [39], [41], [42], [44] and our analyses based on SSU-rDNA plus the two-gene combined trees demonstrate the monophyly of *Moneuplotes* and *Euplotoides*, but reject the monophyly of the other two genera (Figs. 3, S4). Similarly, the three species groups (i.e., single-, double-, or multiple- dargyrome types) defined according to dargyrome patterns (dorsal silverline system) by Gates and Curds [60] are not always monophyletic in molecular phylogenetic trees (our investigation [38], [42]), indicating the presence of more complexity within this group than is evident from dargyrome patterns. Moreover, the three well resolved clades (Clade I-III) repeatedly shown in previous SSU-rDNA trees [38], [39], [41], [42] are not always present in our trees (Figs. 2, 3, S2, S3, S4) nor are they supported by morphological characters, which indicates that these well resolved clades in SSU-rDNA gene trees may not capture valid taxonomic relationships. Finally, clades within Euplotidae are inconsistent with respect to morphology and habit, since the two freshwater species and the marine forms are interdigitated (Figs. 2, 3, S2, S3, S4).

### Evolutionary Patterns in Duplicated Alpha-Tubulin

Among seven euplotid species for which paralogs are detected, duplicated alpha-tubulin genes of all taxa show some changes in the amino acid sequence following duplication (Table 2). Compared to those of *Paramecium tetraurelia*, which has much longer macronuclear chromosomes, there are bigger amino acid distances between paralogs of euplotid species. This elevated level of sequence divergence is similar to patterns in proteins from other ciliates with gene-sized macronuclear chromosomes (Israel et al. 2002, Zufall et al. 2006), and supports the hypothesis that genome processing is associated with increased protein diversification as proposed by previous investigations [30], [61], [62].

## Materials and Methods

No specific permits were required for the described field studies. All locations are not privately-owned or protected in any way, and none endangered or protected species was involved.

### Collection and Identification of Ciliates

We isolated genomic DNAs from 34 morphospecies samples (Fig. 1) from China. Exact collection localities, sample information and GenBank accession numbers of sequenced alpha-tubulin genes are listed in Table 1. All isolates were identified by the methods of Shen et al. [37]. Terminology and systematic classification used in the current paper follow Lynn [31]. The term dargyrome used in the present paper is here defined as in previous reference [38], and refers to the overall geometrical pattern of the dorsal argyrome or “silverline system” in some euplotid ciliates. This pattern consists of net- or web-like structure revealed by silver impregnation methods, which is of great taxonomic importance at generic or specific level [59].

### Extraction and Sequencing of DNA

Genomic DNA was extracted according to methods described in Yi et al. [63]. All DNA samples are extracted from one to several cells of one population, except for that there are two DNA samples for *Diophrys parappendiculata* and *Euplotes sinicus*, which are from two populations, respectively (Table 1). The PCR amplifications of the alpha-tubulin genes were performed using a TaKaRa ExTaq DNA Polymerase Kit (TaKaRa Biomedicals, Japan). Primers used for partial alpha-tubulin gene amplification were Tub-1 (5′-AAG GCT CTC TTG GCGTAC AT-3′) and the reverse primer Tub-2 (5′-TGATGC CTT CAA CAC CTT CTT-3′) [11]. PCR conditions were: 5 min initial denaturation (95°C), followed by 35 cycles of 1 min at 95°C, 1 min at 56°C and 1.5 min at 72°C, with a final extension of 15 min (72°C). The amplicons were directly sequenced using the same primers. However, if paralogs were detected in a sample, then it was purified using the TIANgel Midi Purification Kit and inserted into a pUCm-T vector. Two to nine clones were selected and sequenced by Invitrogen (Shanghai, China). Though it is impossible to detect all paralogs of investigated species due to interpretation of direct sequencing and limited clone samples, these sequences provide an estimate of paralog diversity.

### Data Analyses

Sequence divergence between paralogs of ciliates is not clear. In the present investigation, we follows criterion of previous study [15], which defines sequences that diverge by more than 2% as paralogs, considering sequences errors produced by repeated PCRs and cloning [64]. Under this approach, recent paralogs may be confounded with allelic diversity and some paralogs may be missed, but this should not substantially bias our interpretations.

Five data sets were included in phylogenetic analyses: (1) Atub_n74: alpha-tubulin nucleotide sequences including first two codon positions (74 sequences in total); (2) Atub_aa: alpha-tubulin amino acid (70 sequences in total); (3) Atub-SSU: two-gene combined dataset including all euplotid species available (the paralog with shortest branch length is selected for alpha-tubulin) and other spirotrichean species of Dataset Atub_n74 except for *Discocephalus ehrenbergi* and *Histriculus histrio* for SSU-rDNA, and *D. rotatorius* and *H. cavicola* for alpha-tubulin (52 sequences in total); (4) SSU: SSU-rDNA sequences including all taxa in Dataset Atub-SSU (52 sequences in total); (5)Atub_n52: alpha-tubulin nucleotide sequences with first two codon positions including all taxa in Dataset Atub-SSU (52 sequences in total). For phylogenetic analyses, 27 sequences of alpha-tubulin genes from GenBank were used in addition to ones newly sequenced in the present study. The sequences were aligned using the ClustalW implemented in BIOEDIT 7.0.0 [65], and further modified manually using BIOEDIT. Final alignments used for subsequent phylogenetic analyses included 710 positions (Atub_n74), 355 positions (Atub_aa), 2,303 positions (Atub-SSU) and 1,593 positions (SSU), respectively. GTR + I + C was the best fitted model for nucleotide dataset (Atub_n74) selected by AIC as implemented in MrModeltest v2 [66], and Blosum62+I+G was the best one for amino acid dataset (Atub_aa) selected by AIC as implemented in ProtTest 1.4 [67]. Maximum likelihood analyses, and 1,000 bootstrap replicates, were conducted using RaxML-HPC v7.2.7 [68]. A Bayesian inference (BI) analysis was performed with MrBayes 3.1.2 [69] using the GTR+I+G model selected by MrModeltest 2 [66] under the AIC criterion. Markov chain Monte Carlo (MCMC) simulations were run with two sets of four chains using the default settings: chain length 1,500,000 generations, with trees sampled every 100 generations. The first 3,000 trees were discarded as burn-in. The remaining trees were used to generate a consensus tree and to calculate the posterior probabilities (PP) of all branches using a majority-rule consensus approach. Phylogenetic trees were visualized with TreeView v1.6.6 [70] and MEGA 4 [71].Congruence of different data partitions (in this case genes) was tested with both the incongruence length difference (ILD) test [72] and Shimodaira-Hasegawa (S-H) test [73] as implemented in PAUP*4.0b 10. PAUP* 4.0b 10 was used to generate constraint trees, and resulting trees were compared with unconstrained ML tree using the approximately unbiased (AU) test [74] as implemented in CONSEL package [75].

## Supporting Information

Figure S1 **Identical alpha-tubulin amino acid sites with different nucleotide sequences of** ***Uronychia multicirrus*** **and** ***U. sinica*** **(A);** ***Euplotopsis encysticus*** **and** ***Euplotes*** **cf.** ***antarcticus*** **(B);** ***Euplotes*** **sp.-GZJJM2009121510,** ***Euplotoides parawoodruffi*** **and** ***Euplotopsis*** **sp.-GZJJM2009121508 (C).** A dot indicates a base that is identical to the first species. Solid circles highlight different first codon positions among/between species, and pentagram highlights different second codon position among species.
(TIF)

Figure S2 **Best tree of the Spirotrichea inferred by Maximum likelihood of alpha-tubulin amino acid sequences (Atub_aa).** Species newly sequenced in the present study are shown in bold type. Bootstrap values for branches of the ML tree and posterior probability values for BI tree, respectively, are given on nodes. Fully supported (100%/1.00) branches are marked with solid circles. The scale bar corresponds to 1 substitutions per 100 nucleotide positions. Dargyrome patterns and natural habitats are given after species name of euplotids by symbols.
(TIF)

Figure S3 **Best tree of the Spirotrichea inferred by Maximum likelihood of Dataset Atub_n52. The scale bar corresponds to 1 substitution per 100 nucleotide positions.**
(TIF)

Figure S4 **Best tree of the Spirotrichea inferred by SSU-rDNA sequences (SSU). Bootstrap values for branches of the ML tree is given on nodes.** The scale bar corresponds to 5 substitutions per 100 nucleotide positions. Dargyrome patterns and natural habitats are given after species name of euplotids by symbols.
(TIF)
