# Supplementary figures and images for: Assessing Whether Alpha-Tubulin Sequences Are Suitable for Phylogenetic Reconstruction of Ciliophora with Insights into Its Evolution in Euplotids
Source: PLoS One. 2012 Jul 10;7(7):e40635. doi: 10.1371/journal.pone.0040635 (PMC3393704; doi:10.1371/journal.pone.0040635)

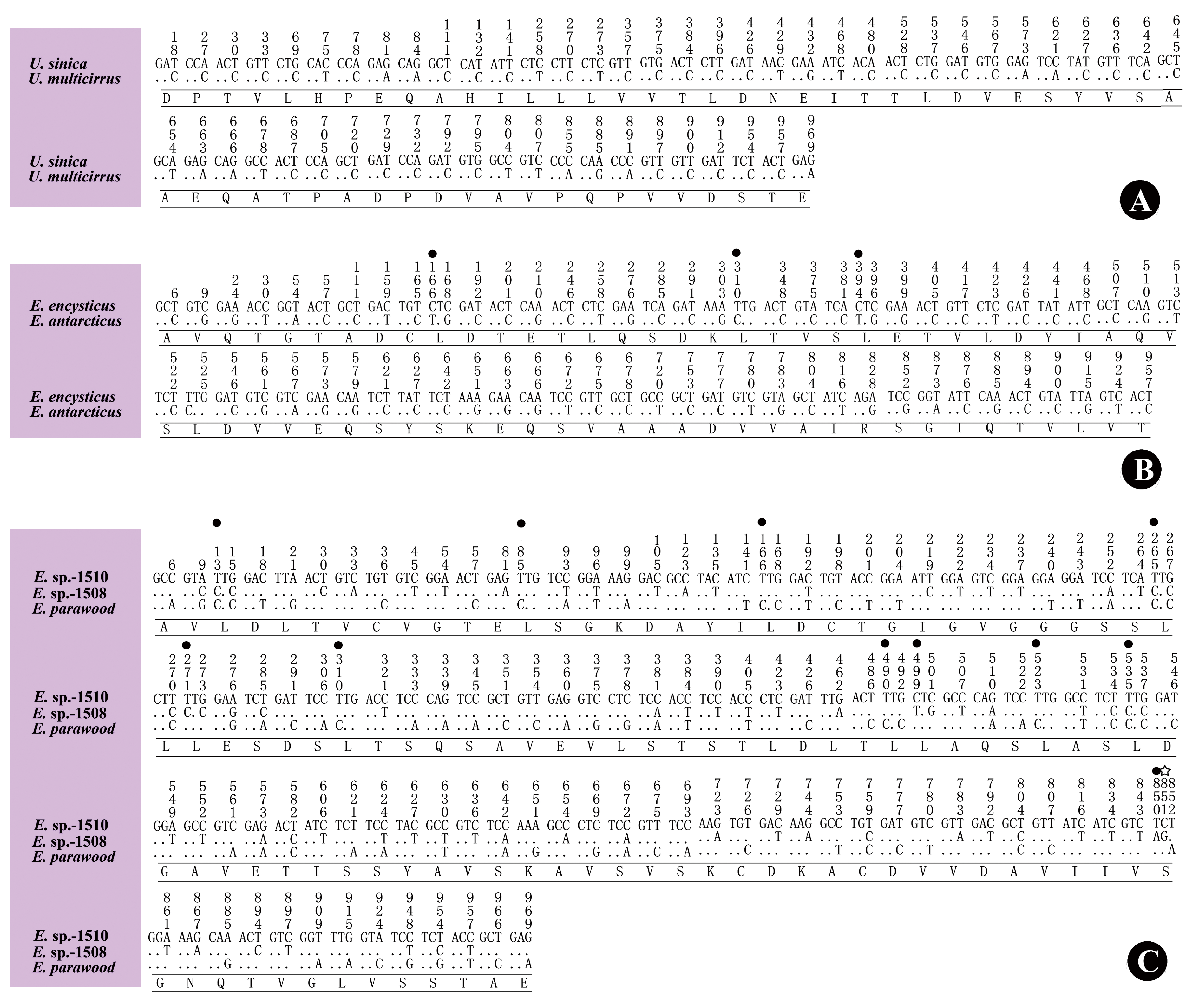

Supplement: Figure S1 — Identical alpha-tubulin amino acid sites with different nucleotide sequences of Uronychia multicirrus and U. sinica (A); Euplotopsis encysticus and Euplotes cf. antarcticus (B); Euplotes sp.-GZJJM2009121510, Euplotoides parawoodruffi and Euplotopsis sp.-GZJJM2009121508 (C). A dot indicates a base that is identical to the first species. Solid circles highlight different first codon positions among/between species, and pentagram highlights different second codon position among species. (TIF) [file pone.0040635.s001.tif]

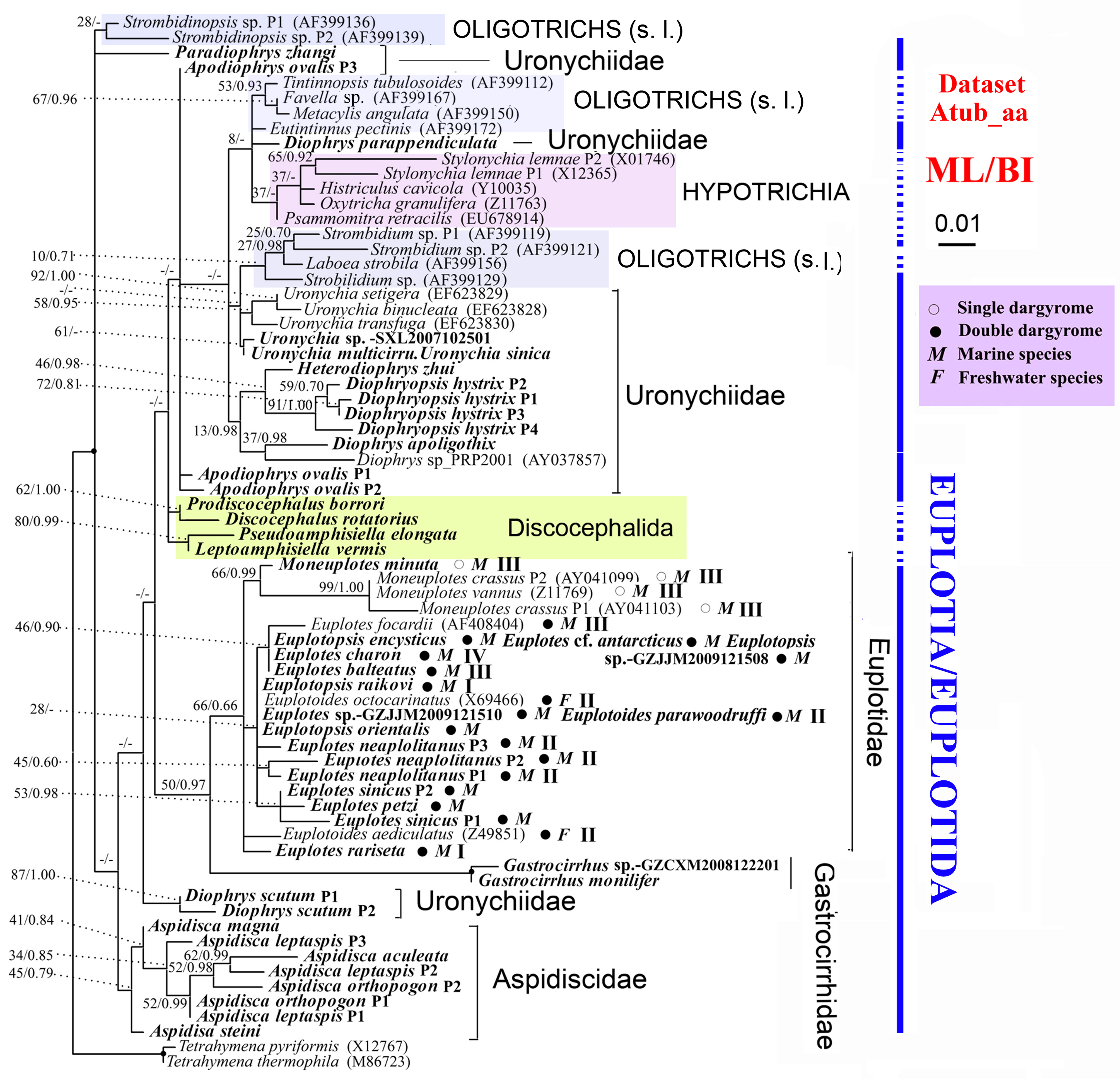

Supplement: Figure S2 — Best tree of the Spirotrichea inferred by Maximum likelihood of alpha-tubulin amino acid sequences (Atub_aa). Species newly sequenced in the present study are shown in bold type. Bootstrap values for branches of the ML tree and posterior probability values for BI tree, respectively, are given on nodes. Fully supported (100%/1.00) branches are marked with solid circles. The scale bar corresponds to 1 substitutions per 100 nucleotide positions. Dargyrome patterns and natural habitats are given after species name of euplotids by symbols. (TIF) [file pone.0040635.s002.tif]

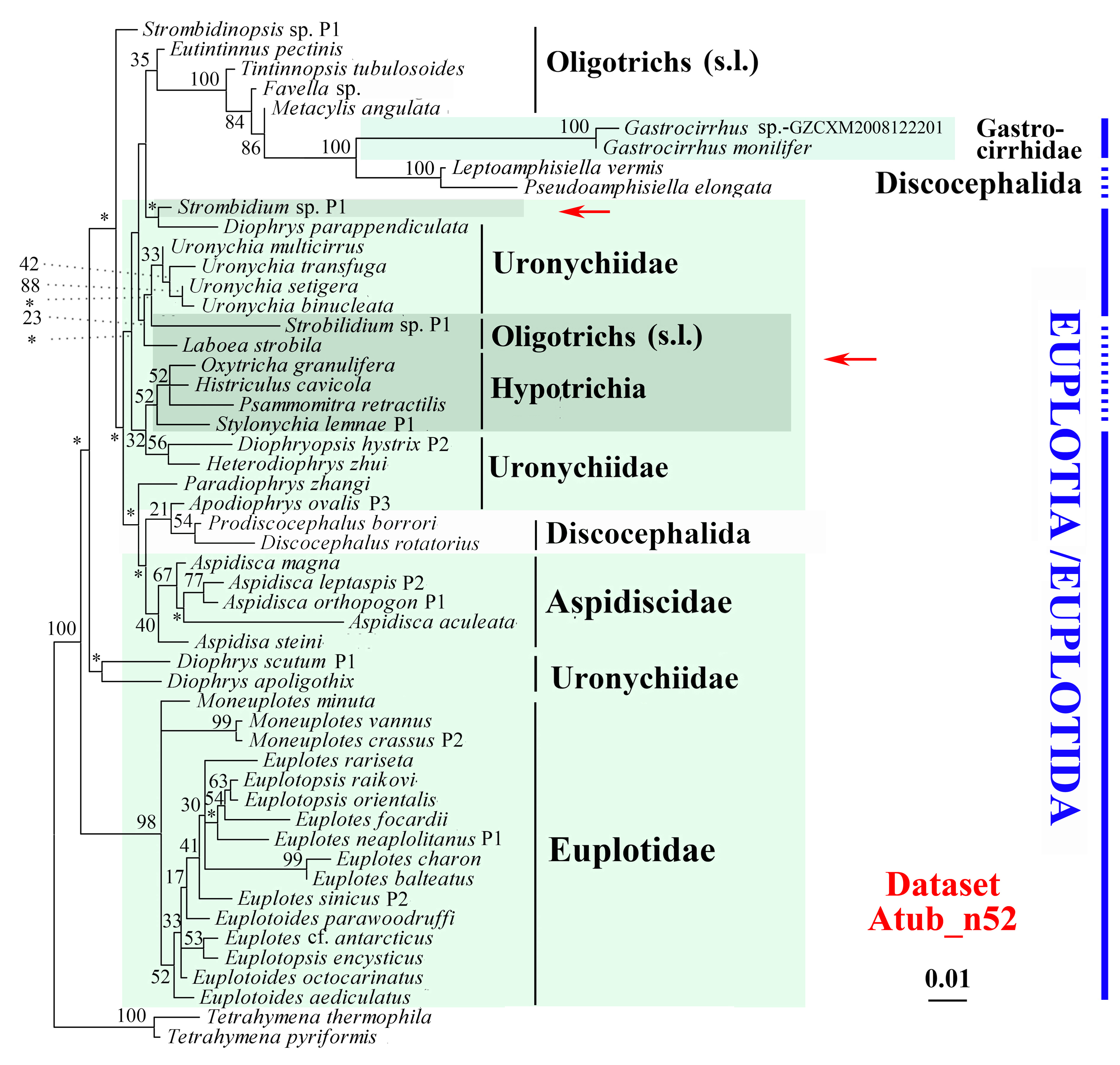

Supplement: Figure S3 — Best tree of the Spirotrichea inferred by Maximum likelihood of Dataset Atub_n52. The scale bar corresponds to 1 substitution per 100 nucleotide positions. (TIF) [file pone.0040635.s003.tif]

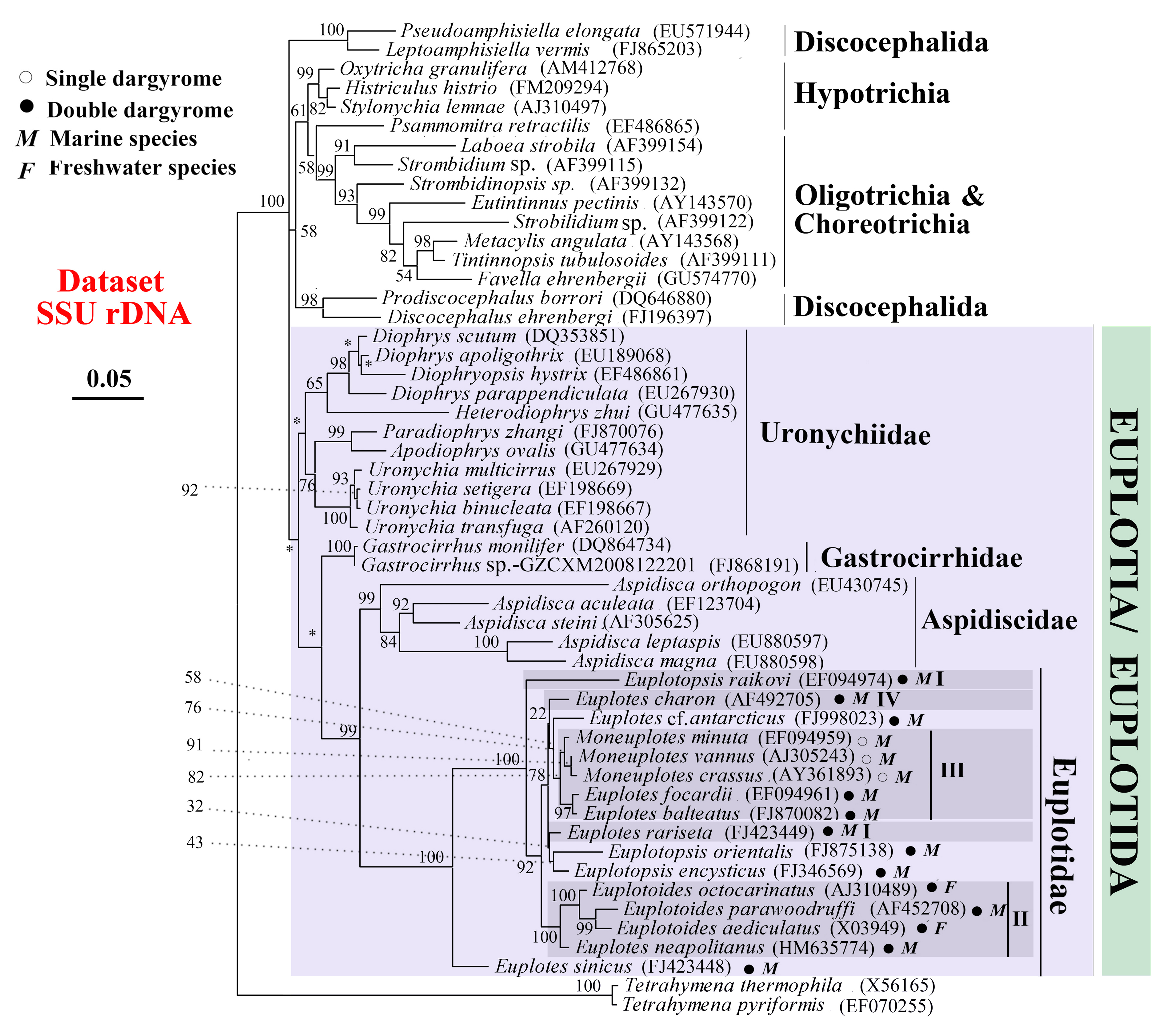

Supplement: Figure S4 — Best tree of the Spirotrichea inferred by SSU-rDNA sequences (SSU). Bootstrap values for branches of the ML tree is given on nodes. The scale bar corresponds to 5 substitutions per 100 nucleotide positions. Dargyrome patterns and natural habitats are given after species name of euplotids by symbols. (TIF) [file pone.0040635.s004.tif]
